# *Salmonella enterica* serovar-specific transcriptional reprogramming of infected cells

**DOI:** 10.1371/journal.ppat.1006532

**Published:** 2017-07-24

**Authors:** Sebastian Hannemann, Jorge E. Galán

**Affiliations:** Department of Microbial Pathogenesis, Yale University School of Medicine, New Haven, Connecticut, United States of America; University of California Davis School of Medicine, UNITED STATES

## Abstract

Despite their high degree of genomic similarity, different *Salmonella enterica* serovars are often associated with very different clinical presentations. In humans, for example, the typhoidal *S*. *enterica* serovar Typhi causes typhoid fever, a life-threatening systemic disease. In contrast, the non-typhoidal *S*. *enterica* serovar Typhimurium causes self-limiting gastroenteritis. The molecular bases for these different clinical presentations are incompletely understood. The ability to re-program gene expression in host cells is an essential virulence factor for typhoidal and non-typhoidal *S*. *enterica* serovars. Here, we have compared the transcriptional profile of cultured epithelial cells infected with *S*. Typhimurium or *S*. Typhi. We found that both serovars stimulated distinct transcriptional responses in infected cells that are associated with the stimulation of specific signal transduction pathways. These specific responses were associated with the presence of a distinct repertoire of type III secretion effector proteins. These observations provide major insight into the molecular bases for potential differences in the pathogenic mechanisms of typhoidal and non-typhoidal *S*. *enterica* serovars.

## Author summary

*Salmonella* Typhimurium and *Salmonella* Typhi are associated with very different clinical presentations. While *S*. Typhimurium causes self-limiting gastroenteritis (i. e. “food poisoning”), *S*. Typhi causes typhoid fever, a systemic, life-threatening disease. The bases for these major differences are not fully understood but are likely to involve many factors. We have compared the transcriptional responses of cultured cells infected with *S*. Typhimurium or *S*. Typhi. We found that these *Salmonella* serovars stimulated distinct transcriptional responses, which could be correlated with their ability to stimulate serovar-specific signal transduction pathways. Importantly, the ability to stimulate these cellular responses was correlated with the presence or absence of specific type III secretion effector proteins. These observations provide major insight into the molecular bases for the differences in the pathogenic mechanisms of typhoidal and non-typhoidal *S*. *enterica* serovars.

## Introduction

*Salmonella enterica* encompasses multiple serovars that are associated with distinct pathogenic features and host specificities [1, 2]. *Salmonella enterica* serovar Typhi (*S*. Typhi), for example, is the cause of typhoid fever, a systemic disease of humans that leads to an estimated 200,000 deaths worldwide [3–6]. In contrast, the broad host *Salmonella* Typhimurium causes limited gastroenteritis (i. e. “food poisoning”) and is one of the most common causes of food-borne illnesses in the industrialized world [1, 2]. The molecular bases for these differences are incompletely understood but they are expected to involve multiple virulence factors that are unique to each serovar. Unique factors to *S*. Typhi include typhoid toxin, which is thought to be responsible for much of the acute specific symptoms associated with typhoid fever [7], and the Vi capsular polysaccharide, which is thought to modulate the inflammatory response to this pathogen [8]. Furthermore, through the process of host adaptation, *S*. Typhi has lost a number of genes that are present in non-typhoidal *Salmonella* serovars [9]. Despite the different clinical presentations, however, *S*. Typhi and *S*. Typhimurium share a substantial portion of their genomes and consequently many pathogenic traits [10–12]. For example, both serovars encode two type III protein secretion systems (TTSSs) within their pathogenicity islands 1 (SPI-1) and 2 (SPI-2), which mediate their close interactions with host cells [13, 14]. Through the activity of the several effector proteins they deliver, these T3SSs mediate bacterial entry, intracellular replication, and the transcriptional reprogramming of the target cells by subverting the cellular machineries that control actin cytoskeleton dynamics, vesicle trafficking, and signal transduction. Despite the highly conserved nature of these T3SSs, the composite of effector protein that they deliver differs among *S*. *enterica* serovars [12]. We have recently shown that even small differences in the T3SS effector protein repertoire translate into profound differences in the biology of different *Salmonella enterica* serovars. For example, the absence of two effector proteins, GtgE and SopD2, which target Rab32, an essential component of a cell-autonomous pathogen restriction pathway, prevents *S*. Typhi replication in non-human hosts [15, 16].

The ability to stimulate transcriptional responses in infected cells is emerging as a central strategy in the pathogenesis of *S*. *enterica* serovars [17–21]. *S*. Typhimurium, for example, stimulates transcriptional responses in intestinal epithelial cells that lead to the production of pro-inflammatory cytokines that initiate the inflammatory response that is central for its pathogenesis [17–21]. Furthermore, this transcriptional re-programming renders the infected cells more permissive for bacterial replication [19]. *S*. Typhi has also been shown to stimulate transcriptional responses in infected cells [22] and infected individuals [20, 23]. Here we have compared the transcriptional responses of cultured epithelial cells infected with *S*. Typhi and *S*. Typhimurium. We have identified serovar specific transcriptional fingerprints that were correlated with the stimulation of specific signal transduction pathways and with the presence or absence of serovar-specific TTSS effector proteins. These findings provide major insight into the molecular bases for the differences in the pathogenic mechanisms of these *S*. *enterica* serovars.

## Results

### *Salmonella* typhimurium and *Salmonella* typhi stimulate specific gene expression patterns in cultured epithelial cells

**Fig 1:**
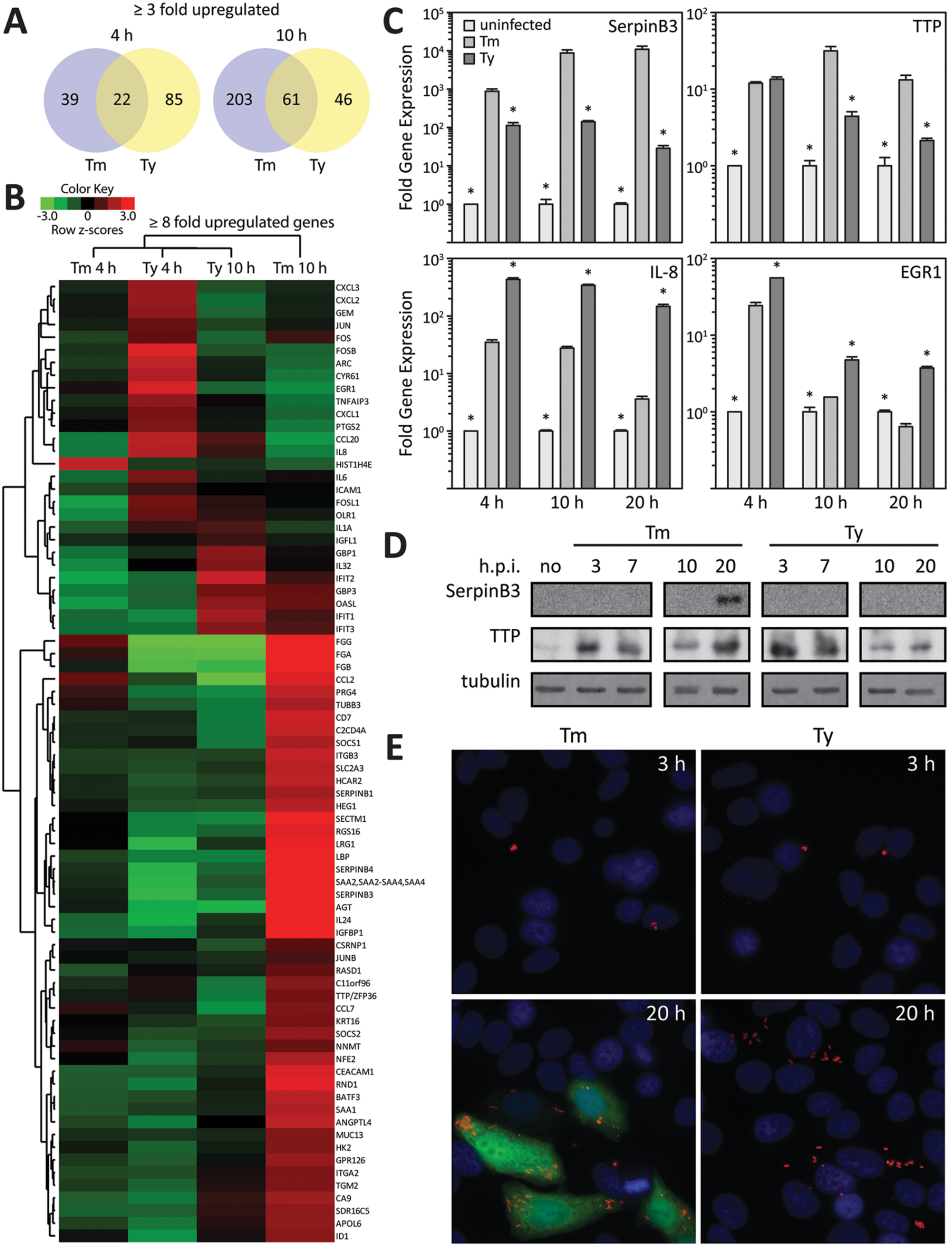
*Salmonella* typhimurium and *Salmonella* typhi stimulate specific gene expression patterns in cultured epithelial cells. (**A**) Venn diagram depicting the number of unique and common genes whose expression changed at least 3 fold at the indicated times after infection with *S*. Typhimurium (Tm) or *S*. Typhi (Ty). (**B**) Heat map of genes whose expression changed at least 8 fold at the indicated times after infection with wild type *S*. Typhimurium (Tm) or *S*. Typhi (Ty). The data are presented as row-normalized heat maps as indicated in the color scale. * p < 0.04, Student t test. (**C**) Expression of selected genes in infected Henle-407 cells at the indicated times after infection. Values (mean ± SD of 3 replicates) represent the GAPDH normalized transcript levels of selected genes in *S*. Typhimurium (Tm) or *S*. Typhi (Ty) infected cultured Henle-407 cells relative to levels of uninfected cells. (**D**) Western blot detection of selected proteins (SerpinB3 and TTP) in infected Henle 407 cells at the indicated time after infection with *S*. Typhimurium (Tm) or *S*. Typhi (Ty). Levels of tubulin in the different samples served as loading controls. (**E**) Immunofluorescence staining of endogenous SerpinB3 in Henle-407 cells after infection with *S*. Typhimurium (Tm) or *S*. Typhi (Ty). Cells were stained at the indicated times after infection with antibodies directed to SerpingB3 (green) or bacterial LPS (red), and DAPI (blue) to detect DNA.

Using RNAseq, we compared the transcriptional responses of cultured epithelial cells infected with wild-type *S*. Typhi, *S*. Typhimurium, or their specific isogenic mutants carrying a deletion in *invA*, which encodes an essential component of the SPI-1 TTSS [24]. We found that cells infected with the wild type strains exhibited distinct transcriptional responses relative to the responses to infection with their respective *invA* mutant strains, which have been previously shown to be similar to mock-infected cells [18, 19]. Four hours after infection, cells infected with *S*. Typhi showed a significant increase (3 fold or higher) in the expression of 107 genes, while 61 genes showed increased expression in cells infected with *S*. Typhimurium. The expression pattern was distinct for each serovar in that only 22 upregulated genes were common to both serovars (Fig 1A and 1B, and S1 Table). The pattern of gene expression in infected cells 10 hs after infection was also distinct for each serovar. At this time point, cells infected with *S*. Typhimurium showed a larger number (264) of upregulated genes than cells infected with S. Typhi (107) (Fig 1A and 1B, and S1 Table), with 61 of the upregulated genes being common to both serovars.

We verified these findings by two alternative approaches. We selected genes whose expression varied in a serotype-specific manner and that previous studies have shown them to be consistently and significantly upregulated after *S*. Typhimurium infection [18, 19] and examined their expression by real time PCR at different times after infection with *S*. Typhimurium or *S*. Typhi. In agreement with the RNA-Seq results, we observed serotype-specific induction of expression of the different genes in infected cells (Fig 1C). For example, genes encoding SerpinB3 or TTP were more highly expressed in *S*. Typhimurium- than in *S*. Typhi-infected cells, while the mRNA levels of IL-8 and EGR1 showed the reverse pattern (Fig 1C). We also examined whether the observed increase in gene expression was reflected in an increase in the respective protein levels by examining the levels of SerpinB3 or TTP in whole cell lysates of *S*. Typhi- or *S*. Typhimruium-infected cells. In line with the expression at the mRNA level, we observed increased levels of TTP during the early course of infection with both serovars (Fig 1D). However, late in infection the TTP levels dropped in *S*. Typhi-infected cells but were maintained in cells infected with *S*. Typhimurium (Fig 1D). Also, consistent with mRNA measurements, SerpinB3 was detectable in *S*. Typhimurium-infected cells late (20 hs) in infection, but was undetectable in cells infected with *S*. Typhi at any time after infection (Fig 1D). These findings were confirmed using immunofluorescence microscopy (Fig 1E). Taken together, these results describe a serovar-specific reprogramming of gene expression in culture epithelial cells after infection with typhoidal (*S*. Typhi) or non-typhoidal (*S*. Typhimurium) *Salmonella enterica* serovars.

### *Salmonella enterica* Stimulates serovar-specific signaling pathways in cultured epithelial cells

**Fig 2:**
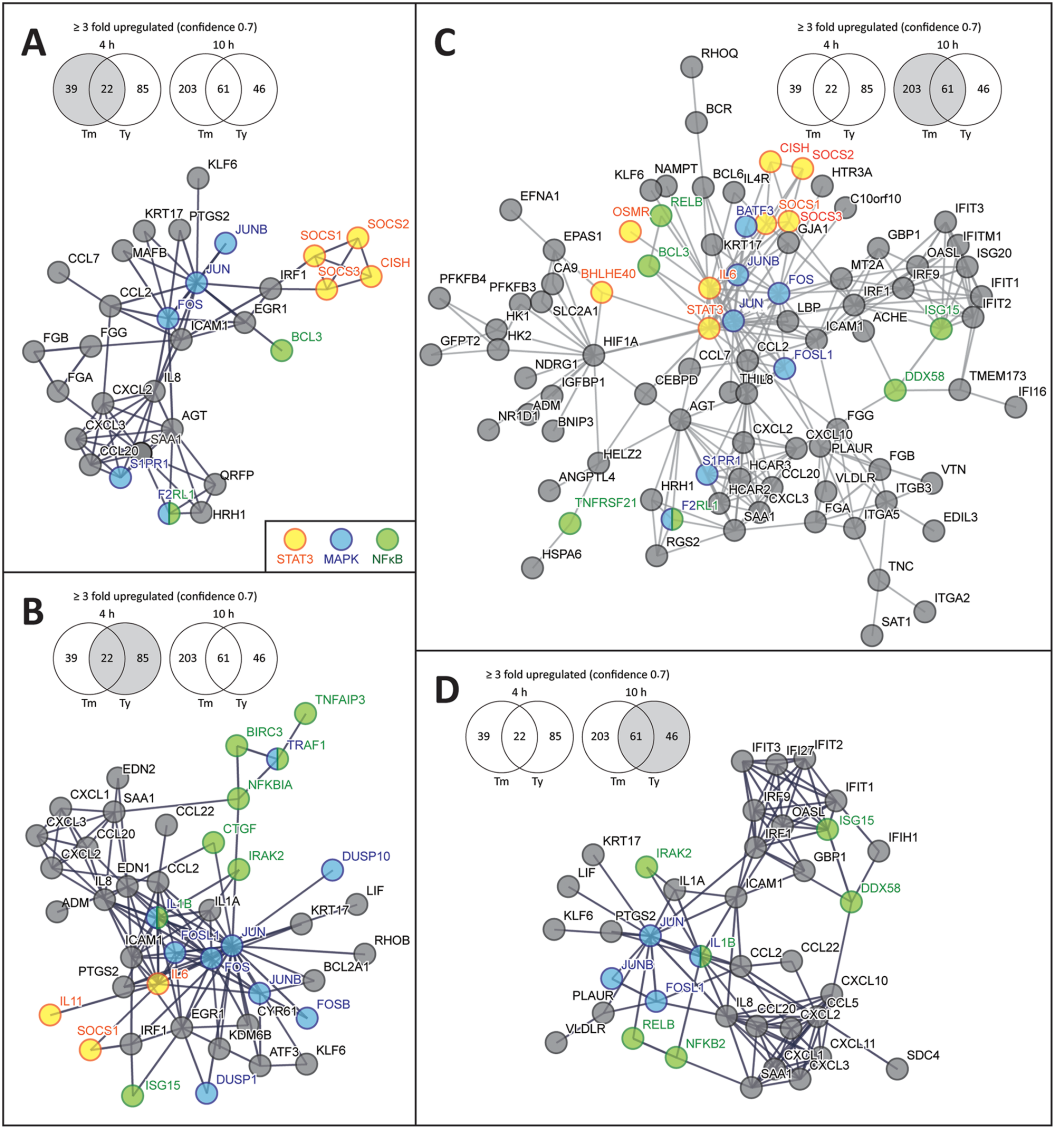
Pathway enrichment of upregulated genes after infection with *S*. Typhimurium or *S*. Typhi. Depicted is the interaction of genes whose expression increased at least 3 fold at 4 h (**A** and **B**) or 10 h (**C** and **D**) after infection with *S*. Typhimurium (**A** and **C**) or *S*. Typhi (**B** and **D**). The analysis was carried out with STRING 10.0 (http://string-db.org/) using high confidence (0.7) parameters. Nodes associated with STAT3, MAPK, or NF-κB signaling are denoted.

**Fig 3:**
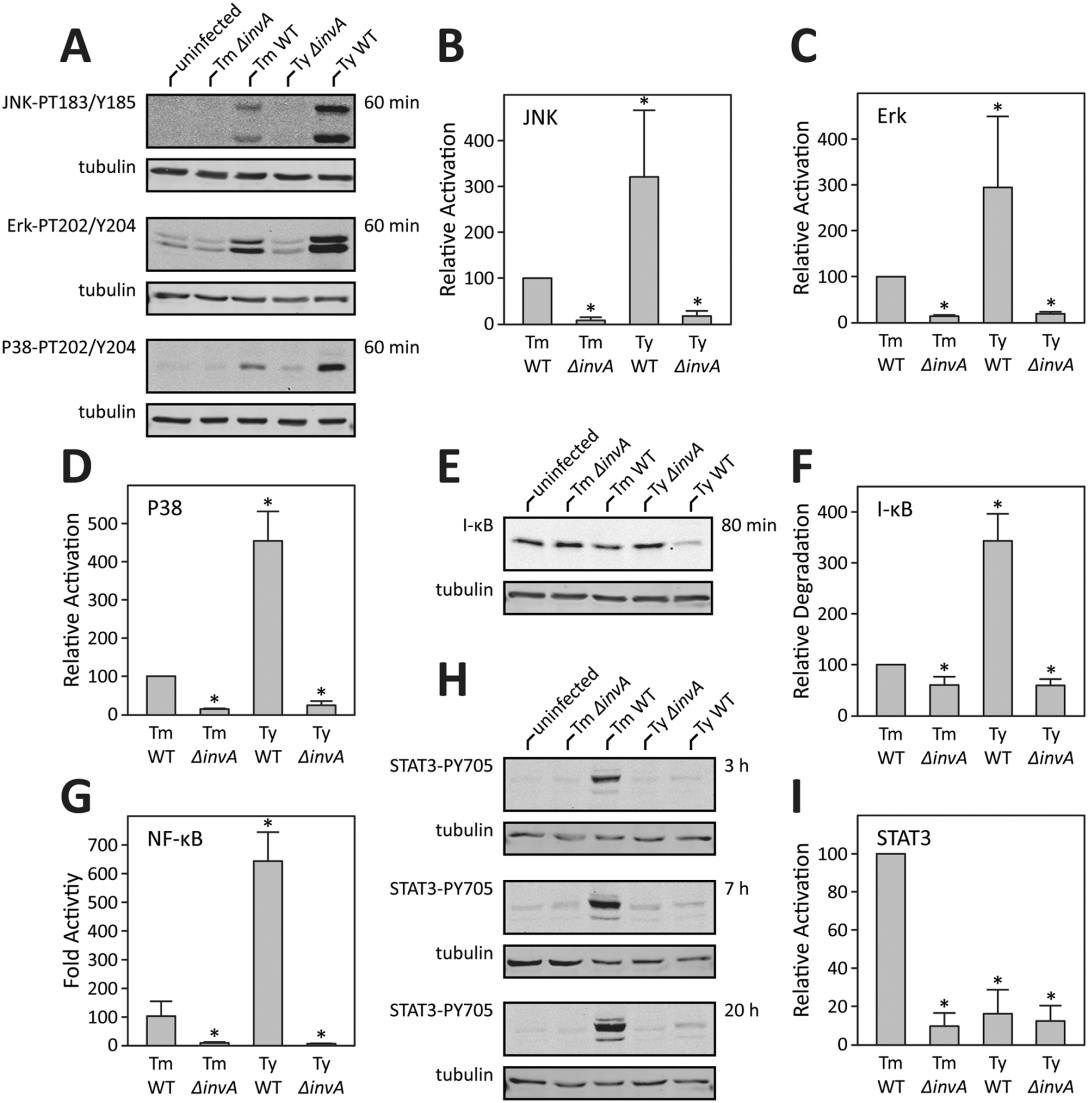
*Salmonella enterica* stimulates serovar-specific signaling pathways in cultured epithelial cells. (**A**-**F**) Stimulation of MAPK and NF-κB signaling after infection with *S*. Typhimurium or *S*. Typhi. Henle-407 cells were infected with *S*. Typhimurium (Ty) or *S*. Typhi (Ty), wild type (WT) or TTSS-deficient (*ΔinvA*) strains for the indicated times and the activation of JNK, Erk, or p38 was assessed by Western blot analysis using antibodies directed to the phosphorylated (activated) forms of these kinases (**A**). Cell lysates were also probed for the levels of I-κB, a measure of NF-κB activation (**E**). Levels of tubulin in the different samples served as loading controls (**A** and **E**). Fold MAPK or NF-κB activation (relative to uninfected cells) was determined by quantifying the signal in the blots with a LI-COR Odyssey imaging system standardizing the signals with the tubulin loading controls. Values are the means (± SD) of at least three independent experiments. *: indicate statistical significance (Students t-test; *p* ≤ 0.01) (**B**-D and **F**). Alternatively, the activation of NF-κB was measured in HEK-293T cells transfected with a NF-κB luciferase reporter construct as indicated above (**G**). Values shown are relative to the activity of the reporter in uninfected control cells and represent the mean ± standard deviation of three independent measurements. (**H** and **I**) Stimulation of STAT3 activation after infection with *S*. Typhimurium or *S*. Typhi. Henle-407 cells were infected as indicated above and the activation of STAT3 was assessed by Western blot analysis using antibodies directed to the phosphorylated (activated) forms of this kinase (**H**). Fold activation (relative to uninfected cells) was determined by quantifying the signal in the blots as indicated above (**I**). Values are the means (± SD) of at least three independent experiments. *: indicate statistical significance (Students t-test; *p* ≤ 0.0001)

The analysis of the pattern of gene expression in cells infected with *S*. Typhi or *S*. Typhimurium for functional associations using the Search Tool for the Retrieval of Interacting Genes/Proteins (STRING) (http://string-db.org) detected serovar-specific networks. Early (4 hs) in infection, the STRING analysis revealed that both *S*. Typhimurium and *S*. Typhi stimulated transcriptional responses associated with the transcription factor NF-κB, as well as those associated with AP1, which is linked to the activation of mitogen-activated protein kinase (MAPK) Erk, Jnk, and p38 signaling (Fig 2) [25–27]. Notably, these responses were more robust in the case of *S*. Typhi-infected cells, resulting in the increased expression of a larger number of host-cell genes associated with these pathways. Later in infection, the STRING analysis indicated a shift in the response to *S*. Typhimurium infection to gene expression patterns associated with STAT3 activation (Fig 2). Importantly, such shift was not apparent in *S*. Typhi-infected cells, which exhibited a more restricted response with a pattern of gene expression more consistent with the activation of NF-κB signaling pathways (Fig 2). The distinct pattern of gene expression observed in cells infected with the different *S*. *enterica* serovars suggested that *S*. Typhi and *S*. Typhimruium stimulate distinct signaling pathways. Previous studies in our laboratory have shown that *S*. Typhimurium stimulates MAPK, NF-κB, and STAT3 signaling pathways in cultured epithelial cells [17–19, 28]. However, equivalent studies have not been carried out with *S*. Typhi. To gain insight into the mechanisms underlying the distinct transcriptional responses observed after infection with *S*. Typhimurium and *S*. Typhi, we examined the stimulation of MAPK, NF-κB, and STAT3 signaling pathways in cultured epithelial cells. Consistent with previous studies [17, 18, 28], infection with *S*. Typhimurium resulted in increased phosphorylation of the MAPKs Erk, Jnk, and p38 as well as in the activation of NF-κB as indicated by the degradation of I-κB, an inhibitor of NF-κB, and the activation of a luciferase reporter (Fig 3A–3G). In addition and also consistent with previous observations [19], *S*. Typhimurium infected cells, particularly later in infection, showed potent activation of STAT3 as monitored by the phosphorylation of STAT3^Y705^ (Fig 3H and 3I). In contrast, STAT3 phosphorylation was not detected in *S*. Typhi-infected cells throughout infection (Fig 3H and 3I). Furthermore, relative to *S*. Typhimurium-infected cells, *S*. Typhi-infected cells showed a more robust activation of NF-κB and MAPK signaling (Fig 3A–3G). These results are consistent with the STRING analysis of the pattern of gene expression, which showed an enrichment of genes associated with the stimulation of these signaling pathways in *S*. Typhi-infected cells. Taken together these findings indicate that *S*. Typhi and *S*. Typhimurium stimulate distinct signaling pathways that lead to a distinct pattern of host cell gene expression.

### Type III secretion effector proteins impart specificity to the cellular responses to *S*. *enterica* serovars typhi and typhimurium

**Fig 4:**
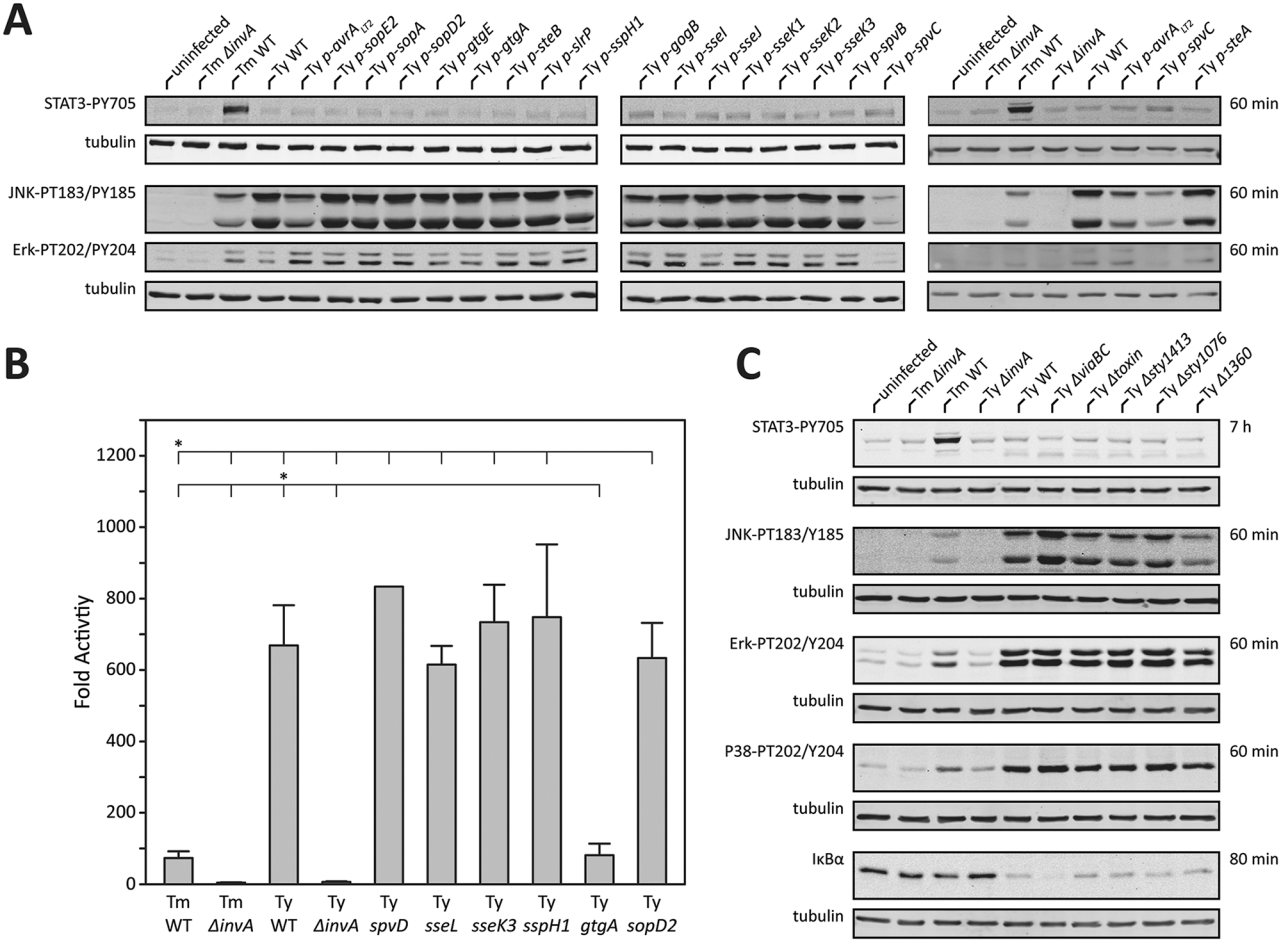
Type III secretion effector proteins impart specificity to the cellular responses to *S*. *enterica* serovars Typhi and Typhimurium. (**A** and **B**) Stimulation of MAPK, STAT3 and NF-κB activation after infection with *S*. Typhi (Ty) strains expressing the indicated *S*. Typhimurium effector proteins. Henle-407 cells were infected with the indicated *S*. Typhimurium (Ty) or *S*. Typhi (Ty) strains and the activation of JNK, Erk, or STAT3 was assessed by Western blot analysis using antibodies directed to the phosphorylated (activated) forms of these kinases (**A**). Alternatively, the activation of NF-κB in infected cells was measured in HEK-293T cells transfected with a NF-κB luciferase reporter construct (**B**). Values shown are relative to the activity of the reporter in uninfected control cells and represent the mean ± standard deviation of three independent measurements. *: indicate statistical significance (Students t-test; *p* ≤ 0.0001) **(C**) Stimulation of MAPK, STAT3 and NF-κB activation after infection with different mutant strains of *S*. Typhi (Ty). Henle-407 cells were infected with the indicated *S*. Typhi (Ty) deletion mutants and the activation of JNK, Erk, p38, or STAT3 kinases was assessed by Western blot analysis using antibodies directed to the phosphorylated (activated) forms of these kinases. Cell lysates were also probed for the levels of I-κB, a measure of NF-κB activation.

As shown above, cells infected with different *Salmonella enterica* serovars exhibited distinct signaling responses that resulted in distinct patterns of gene expression. To gain insight into the molecular bases for the observed differences, we sought to identify serovar-specific genes that might be correlated with the different responses. It is well established that the ability of *Salmonella* to interact with host cells is strictly dependent on the activity of effector proteins delivered through its SPI-1 and SPI-2-encoded type III secretion systems [13]. Furthermore, our analysis revealed that the transcriptional and signaling responses to both, *S*. Typhi and *S*. Typhimurium were dependent on a functional type III secretion system (Figs 1 and 3). Although the components of these systems are highly conserved across all *Salmonella* serovars, the effector proteins they deliver are not, and different *Salmonella* serovars encode a specific composite of effectors [12]. Notably, in comparison to *S*. Typhimurium, *S*. Typhi encodes a reduced number of effectors, which is consistent with the genome reduction driven by its adaptation to the human host [29]. We therefore reasoned that at least some of the differences observed in the signaling capacity of these two *Salmonella* serovars might be due to differences in the effector protein repertoire. To test this hypothesis, we constructed a series of *S*. Typhi strains encoding each one of the specific effectors that are present in *S*. Typhimurium but that are absent from *S*. Typhi. We then examined the capacity of these different *S*. Typhi strains to activate Jnk, Erk, or STAT3 signaling. We found that expression of the *S*. Typhimurium effector proteins AvrA or SpvC reduced the ability of *S*. Typhi to activate the MAPKs Jnk and Erk to levels that were equivalent with those observed in *S*. Typhimurium-infected cells (Fig 4A). These findings are consistent with previous studies that have shown that AvrA negatively modulates the ability of *S*. Typhimurium to activate Jnk by inhibiting its activating kinase MKK7 [30]. Likewise, these findings are also consistent with previous reports indicating that SpvC is a threonine lyase that directly targets and inhibits Erk kinases [31]. These results therefore indicate that the absence of AvrA and SpvC in *S*. Typhi results in a heightened ability to stimulate MAPK signaling.

To gain insight into the mechanisms underlying the increased ability of *S*. Typhi to stimulate NF-κB activation, we investigated the effect of expressing *S*. Typhimurium T3SS-effector proteins that have been previously shown to have the capacity to modulate this signaling pathway. Expression of *spvD*, which encodes a cysteine protease that has been reported to negatively regulate nuclear transport of NF-κB components [32, 33], did not affect the ability of *S*. Typhi to stimulate NF-κB signaling (Fig 4B). Similarly, expression of *ssek3*, *sseL*, or *sspH1*, which have been reported to modulate NF-κB signaling [34–36], had no effect on the ability of *S*. Typhi to stimulate the expression of a NF-κB dependent reporter (Fig 4B). Recent studies from our laboratory identified a family of highly related effector proteins, GtgA, GogA and PipA, which redundantly inhibit NF-κB by proteolytically targeting the RelA and RelB transcription factors [37]. *S*. Typhi does not encode homologs of GtgA or GogA although it encodes a homolog of PipA. However, we were unable to detect expression of *pipA* suggesting that at least under the experimental conditions used here, this gene is not expressed in *S*. Typhi. We expressed GtgA, one of the members of this family of effectors, and examined its effect on the ability of *S*. Typhi to stimulate NF-κB signaling. Consistent with previous studies in *S*. Typhimurium, expression of GtgA markedly diminished the ability of *S*. Typhi to stimulate the expression of an NF-κB reporter (Fig 4B). These results therefore indicate that, similar to MAPK signaling, the absence in *S*. Typhi of specific effectors with inhibitory activity results in a heightened ability to stimulate NF-κB.

Previous studies have shown that the ability of *S*. Typhimurium to stimulate MAPK, NF-κB, and STAT3 signaling pathways is dependent on the functionally redundant activities of the SPI-1 T3SS effectors proteins SopE, SopE2 and SopB [18, 19, 38]. While *sopE* and *sopB* are highly conserved, in *S*. Typhi *sopE2* has a frame-shifting mutation that leads to a non-functional polypeptide [29]. The absence of one of the effectors responsible for the activation of STAT3 suggested the possibility that such a loss could account for the inability of *S*. Typhi to activate this signaling pathway. However we found that the expression of *sopE2* in *S*. Typhi did not confer the ability to stimulate STAT3 activation (Fig 4A). In fact, there was no detectable effect on the ability of *S*. Typhi to stimulate STAT3 phosphorylation after heterologous expression of any of the *S*. Typhimurium effectors that are absent from this serovar (Fig 4A). Given the conservation of the effectors responsible for STAT3 activation, we hypothesized that *S*. Typhi may encode an as yet unidentified effector protein, absent from *S*. Typhimurium, that inhibits STAT3 activation. We therefore searched the *S*. Typhi genome for genes encoding putative TTSS effector proteins that are absent from *S*. Typhimurium. We identified three open reading frames, *sty1423*, *sty1360*, and s*ty1076*, which encode homologs of the *E*. *coli* type III secreted effectors EspN [39], OspB [40], and NleG [39], respectively. We found that the deletion of any of these genes had no impact in the ability of *S*. Typhi to stimulate signal transduction pathways, and more specifically, none of the *S*. Typhi deletion mutants were able to stimulate STAT3 activation (Fig 4C). While *S*. Typhimurium and *S*. Typhi share their core genome, there are a number of virulence factors that are uniquely present in *S*. Typhi [11]. One of the virulence factors unique to *S*. Typhi is the Vi antigen, a capsular polysaccharide encoded within its SPI-7 pathogenicity island that has been proposed to interfere with Toll receptor agonists [8]. However, deletion of *viaA* and *viaB*, which encode essential enzymes for the synthesis of Vi antigen, had no effect on the ability of *S*. Typhi to stimulate STAT3 signaling although it had a small but measurable enhancement of its ability to stimulate NF-κB signaling (Fig 4C). Similarly, removal of typhoid toxin, a critical *S*. Typhi virulence factor [7], had no effect on its ability to stimulate any of these signaling pathways including STAT3 (Fig 4C). These results indicate that an as yet unidentified factor may inhibit the ability of S. Typhi to stimulate STAT3 signaling.

In summary, we have identified specific effector proteins that provide mechanistic explanations for some of the differences in the signalling pathways stimulated by *S*. Typhimurium and *S*. Typhi, which lead to distinct host cell transcriptional responses to these two pathogens. The absence of these effector proteins from *S*. Typhi suggest that the process of host-adaptation may have driven differences in the ability of these serovars to stimulate specific transcriptional responses, which may impact disease.

## Discussion

**Fig 5:**
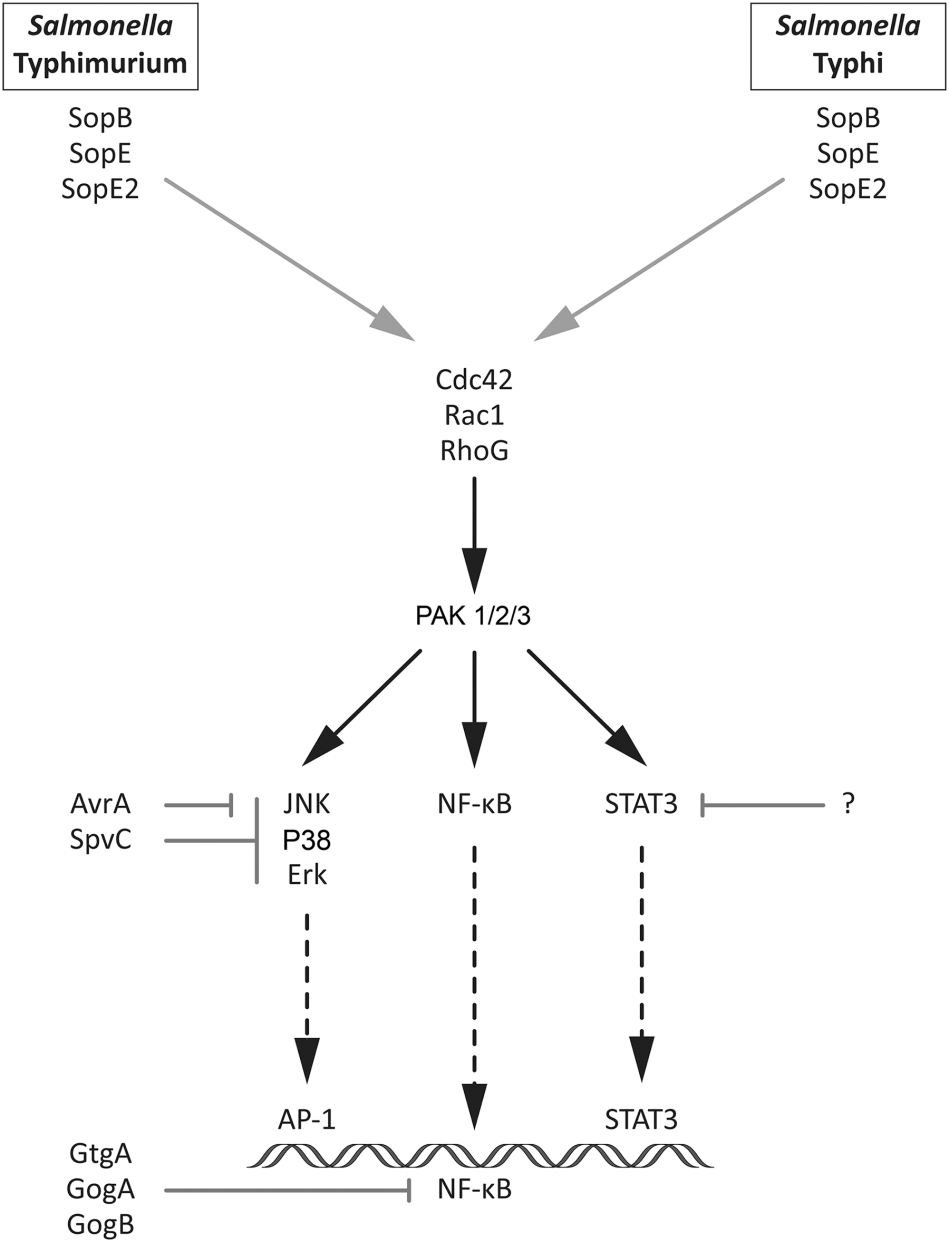
Model for the mechanism of stimulation of transcriptional responses by *S*. Typhimurium and *S*. Typhi.

The ability to stimulate transcriptional responses is thought to be central for the pathogenesis of many microbial pathogens [26]. In the case of *S*. Typhimurium, transcriptional responses in intestinal epithelial cells are central to its capacity to stimulate inflammation in the gut epithelium [17, 18, 28, 41, 42], which is instrumental for its ability to acquire essential nutrients and compete with the resident microbiota [43, 44]. Furthermore, previous work from our laboratory has shown that the ability of *S*. Typhimurium to re-program gene expression is also important for its replication within epithelial cells [19]. *S*. Typhi has also been shown to re-program gene expression in cultured cells [22] and in infected individuals [20, 23], although the significance of these observations for its pathogenic mechanisms remains to be established. The stimulation of transcriptional responses in infected cells by *Salmonella* is strictly dependent on the presence of the SPI-1-encoded TTSS, which through the delivery of specific effectors stimulate signal transduction pathways leading to re-programming of gene expression [17, 28]. More specifically, the delivery of the effector proteins SopE, SopE2, and SopB, results in the direct activation of Rho-family GTPases and the subsequent stimulation of downstream signaling, which ultimately results in host-cell transcriptional reprogramming [18, 38] (Fig 5). In this study, we have compared the transcriptional response of cultured epithelial cells after infection with *S*. Typhimurium and *S*. Typhi. We found that each serovar stimulates a distinct pattern of gene expression associated with specific signaling events. The transcriptional profile of cells infected with *S*. Typhi is consistent with the activation of MAPK and NF-κB signaling pathways, both early and late in infection. The transcriptional profile of cells infected with *S*. Typhimurium was also consistent with the activation of MAPK and NF-κB signaling pathways, particularly early in infection. However, late in infection and in sharp contrast to cells infected with *S*. Typhi, cells infected with *S*. Typhimurium showed a pattern of gene expression associated with the activation of STAT3 signaling. We found that these transcriptional responses were entirely consistent with the signaling pathways stimulated by these pathogens. Both *S*. Typhi and *S*. Typhimurium-infected cells showed activation of NF-κB and MAPK signaling pathways, particularly early in infection. However, the level of activation was clearly more robust in the case of cells infected with *S*. Typhi. Cells infected with *S*. Typhimurium showed a marked activation of STAT3, particularly late in infection. In contrast, cells infected with *S*. Typhi showed no detectable activation of this signaling pathway. The pattern of signaling responses in S. Typhi-infected cells with a heightened activation of NF-κB and MAPK signaling pathways, traditionally associated with inflammatory responses, is surprising considering that S. Typhi infections are known to result in less intestinal inflammation that infections with S. Typhimurium [1–6]. However, S. Typhi infections (i. e. typhoid fever) do results in higher fever, another marker of an inflammatory response. Furthermore, it is well established that transcriptional responses are profoundly affected not only by the nature of the signaling pathways but also by the strength and duration of signaling [45, 46]. In this context, the substantially different pattern of gene expression observed in cells infected with *S*. Typhi and *S*. Typhimurium is entirely consistent with the difference in the nature, strength, and duration of the signaling responses stimulated by these pathogens. Nevertheless, more studies will be required to determine how and if the different transcriptional and signaling responses stimulated by these pathogens contribute to the differences observed in their clinical presentations.

The ability of *Salmonella* to stimulate signaling pathways is dependent on the functionally redundant activity of the TTSS effector proteins SopE, SopE2, and SopB. Given the conservation of these effector proteins across *Salmonella* serovars, it was surprising to observe differences in the signaling responses to *S*. Typhi and *S*. Typhimurium in infected cells. Our results indicate that the heightened MAPK and NF-κB activation observed in *S*. Typhi infected cells is most likely not due to differences in the agonistic capacity of these two *Salmonella* serovars but rather to differences in their ability to down-modulate those responses after their stimulation (Fig 5). Indeed, we found that the absence in *S*. Typhi of effector proteins that in *S*. Typhimurium antagonize MAPK and NF-κB signaling pathways account at least in part for the unique responses that follow infection with each one of these pathogens. More specifically, we found that expression of AvrA, SpvC, and GtgA, which target the MAPK and NF-κB signaling pathways, reduced the level of activation of these pathways to a level comparable to that observed in cells infected with *S*. Typhimurium. In contrast, no *S*. Typhimurium effector protein was able to confer upon *S*. Typhi the ability to stimulate STAT3 signaling. Since the stimulation of STAT3 signaling by *S*. Typhimurium is strictly dependent on the effectors proteins SopE and SopB [19], which are highly conserved in *S*. Typhi, these observations suggest the presence of an as yet unidentified factor in *S*. Typhi that may inhibit STAT3 signaling.

It is becoming increasingly clear that even small differences in the repertoire of TTSS effector proteins in different *Salmonella enterica* serovars can have a profound effect in the manner in which these pathogens interact with their hosts. For example, the absence of just two effector proteins, GtgE and SopD2, can have a very significant impact on the ability of the human-adapted *S*. Typhi to explore other hosts [15, 16]. These two effectors target Rab32, which coordinates the activity of a cell-autonomous pathogen restriction mechanism. We have shown here another example in which differences in the composite of T3SS effector proteins in *S*. Typhi and *S*. Typhimurium can have a significant impact in their ability to reprogram gene expression in infected epithelial cells. How these differences may affect disease is not clear but it is known that *S*. Typhi and *S*. Typhimurium interaction with the gut epithelium leads to markedly different outcomes. While *S*. Typhimurium stimulates an acute, though self-limiting, inflammatory response leading to diarrhea, *S*. Typhi intestinal infection leads to the invasion of deeper tissue with little to no diarrhea. It is possible that the differences in the ability of these pathogens to stimulate signaling pathways leading to different patterns of gene expression in intestinal epithelial cells may account for some of the observed differences in the pathogenesis of these two *Salmonella* serovars.

## Materials and methods

### Bacterial strains, growth conditions, cDNA constructs, and other reagents

All bacterial strains used in this study were derived from the *Salmonella enterica* serovar Typhimurium strain SL1344 [47] or *Salmonella enterica* serovar Typhi strain ISP2825 [48] and are listed in S2 Table. Plasmids used in this study are listed in S3 Table and have either been described before or were constructed as part of this study using standard recombinant DNA techniques. *S*. Typhimurium and *S*. Typhi were grown under conditions that increase expression of the SPI-1 T3SS [49]. Briefly, a 1:20 dilution of a bacterial overnight cultures were grown at 37°C in L-broth containing 0.3 M NaCl until an OD_600_ = 0.9 prior to their use in infections. Antibodies and other reagents were purchased from the indicated companies: rabbit-anti-TTP (Abcam); rabbit-anti-*Salmonella* O Group B Antiserum (Becton Dickson); rabbit-anti- Phospho-STAT3 (Ser727), rabbit-anti-Phospho-STAT3 (Tyr705), Erk (Thr202, Tyr204), JNK (Thr183, Tyr185), P38 (Thr202, Tyr204) and I-kBα (Cell Signaling Technology); mouse-anti-SerpinB3 (Santa Cruz Biotechnology); mouse-anti-tubulin (Sigma-Aldrich); secondary antibodies (Molecular Probes); 49,6-diamidino-2-phenylindole (DAPI) (Sigma-Aldrich).

### Cell culture, transfections, bacterial infections and luciferase reporter assay measurments

The human embryonic kidney epithelial HEK 293T (ATCC) and and epithelial Henle-407 (Roy Curtiss laboratory collection) cell lines were cultured in antibiotic free Dulbecco’s Modified Eagle Medium (DMEM, Gibco) supplemented with 10% bovine calf (Henle-407) or bovine fetal (HEK-293T) sera. For bacterial infections, serum-starved (DMEM without serum) Henle-407 cells at a confluency of 80% were washed with pre-warmed Hank’s buffered salt solution (HBSS) and allowed to equilibrate in HBSS for 15 min at 37°C. Cells were then infected for 1 h with *S*. Typhimurium or *S*. Typhi strains at an adjusted multiplicities of infection (MOI) so as to insure equal number of internalized bacteria as indicated in the figure legends. Thus due to the slightly reduced infection rate of *S*. Typhi, infections were done with a 5-fold excess relative to *S*. Typhimurium (S1 Fig). In all *S*. Typhimurium infections an MOI of 20 was used except for experiments involving measurement of gene expression by PCR (MOI of 30), immunofluorescence (MOI = 5) or Luciferase measurements (MOI of 10). Infected cells were washed once with pre-warmed PBS and incubated for 1 h in pre-warmed DMEM containing 50 μg/ml gentamicin. Cells were washed again with PBS and further incubated in pre-warmed DMEM containing 10 μg/ml gentamicin for the indicated times. Similarly, HEK-293T cells were seeded in a 24-well plate and transfected with the NFκB reporter plasmid as previously described [37]. Transfected cells were serum-starved 18 hs prior to infection with the different *S*. Typhimurium or *S*. Typhi strains as described above and harvested 7 hs post infection for measurement of luciferase activity using a Dual luciferase reporter assay (Promega).

### Statistical analysis

Statistical significance was calculated by a two-tailed distributed paired Student’s t-test with equal variance. Resulting *p* values of less than 0.05 were considered significantly different.

### Western blotting

Cells were washed once with PBS, lysed in 2 x SDS Laemmli buffer and boiled for 10 min. Proteins in cell lysates were separated by SDS-PAGE, transferred to nitrocellulose membranes. Membranes were washed once with Tris buffered saline (TBS), blocked in a buffer containing 3% BSA or 5% milk in TBS for 30 min at room temperature and probed with the respective primary and secondary antibodies in blocking solution supplemented with 0.02% SDS and 0.1% Tween 20. Blots were visualized and analyzed using the Odyssey LI-COR system and the LI-COR Odyssey application software. Alternatively, blots were visualized by enhanced chemiluminescence (ECL).

### Immunofluorescence microscopy

Human epithelial cells grown on glass coverslips were infected with *S*. Typhimurium or *S*. Typhi strains as described above, washed once with HBSS and fixed in 4% PFA/PBS for 15 min at RT. Cells were treated with blocking solution (3% BSA, 0.1% Saponin, 50 mM NH4Cl in PBS) for 20 min at RT, probed with primary antibody overnight at 4°C in a wet chamber, washed three times in blocking solution and subsequently probed with secondary antibody in combination with 4,6-Diamidino-2-phenylindole, dihydrochloride (DAPI) for 30 min at RT. Finally, glass coverslips were washed twice with blocking solution, PBS and water before they were mounted on glass slides and examined by epifluorescence microscopy (Nikon Diaphot) and the Micro-Manager software [50].

### Quantitative real-time PCR

RNA isolation, *in vitro* transcription and quantitative real-time PCR were carried out as described elsewhere [18]. Briefly, total RNA from serum starved and infected Henle-407 cells was isolated using the ‘‘RNeasy Mini Kit” (QIAGEN). Following DNAse treatment, RNA was transcribed using the iScript reverse transcriptase (BIO RAD). Transcript levels were determined in an iCycler real time PCR machine (BIO RAD) using gene specific primer sets (S4 Table), which have been designed by PrimerBank (http://pga.mgh.harvard.edu/primerbank/).

### RNA-Seq

Total RNA was isolated from infected, serum starved Henle-407 cells at the indicated time points as described for quantitative real-time PCR. Samples were submitted to the Yale University’s Center for Genomic Analsysis on an Illumina HiSeq 2500 system. The sequencing data was analyzed using the Galaxy platform [51] (http://www.usegalaxy.org) with the TopHat package for alignment of cDNA fragments in combination with mapping to the human Hg19 reference genome and the Cufflinks package to estimate differential transcript abundance applying a false discovery rate (FDR) of 0.05 [http://www.nature.com/nprot/journal/v7/n3/full/nprot.2012.016.html]. For further analysis, only genes above 150 nucleotides were considered and the fold differences in transcript abundance calculated.

## Supporting information

S1 Fig Equivalent *S*. Typhimurium and *S*. Typhi internalization into cultured epithelial cell after infection with adjusted multiplicity of infection. Cultured Henle-407 epithelial cells were infected with wild type S. Typhimurium or S. Typhi or its type III secretion deficient isogenic *invA* mutants at a multiplicity of infection of 20 (for *S*. Typhimurium strains) or 100 (for *S*. Typh strains). Bacterial internalization was measured by the gentamicin-protection assay.
(DOCX)

S1 Table List of genes whose expression changed at least 3 fold after infection with either S. Typhimurium or S. Typhi. (PDF)

S2 Table List of strains used in this study. (PDF)

S3 Table List of plasmids used in this study. (PDF)

S4 Table qRT-PCR primers used in this study. (PDF)
