# Supplementary material for: Salmonella enterica serovar-specific transcriptional reprogramming of infected cells
Source: PLoS Pathog. 2017 Jul 24;13(7):e1006532. doi: 10.1371/journal.ppat.1006532 (PMC5549772; doi:10.1371/journal.ppat.1006532)
Supplement: S1 Fig — Cultured Henle-407 epithelial cells were infected with wild type S. Typhimurium or S. Typhi or its type III secretion deficient isogenic invA mutants at a multiplicity of infection of 20 (for S. Typhimurium strains) or 100 (for S. Typh strains). Bacterial internalization was measured by the gentamicin-protection assay. (DOCX) [file ppat.1006532.s001.docx]

**
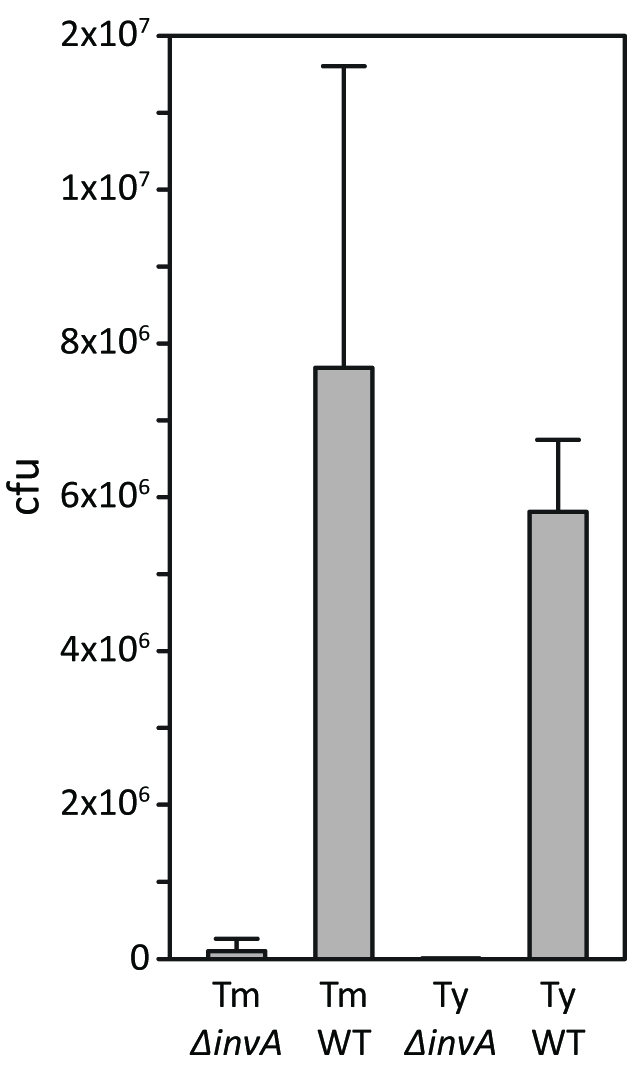
**

**S1 Fig.** Equivalent S. Typhimurium and S. Typhi internalization into cultured epithelial cell after infection with adjusted multiplicity of infection. Cultured Henle-407 epithelial cells were infected with wild type S. Typhimurium or S. Typhi or its type III secretion deficient isogenic *invA* mutants at a multiplicity of infection of 20 (for *S.* Typhimurium strains) or 100 (for *S.* Typh strains). Bacterial internalization was measured by the gentamicin-protection assay.
