# Supplementary material for: Salmonella enterica serovar-specific transcriptional reprogramming of infected cells
Source: PLoS Pathog. 2017 Jul 24;13(7):e1006532. doi: 10.1371/journal.ppat.1006532 (PMC5549772; doi:10.1371/journal.ppat.1006532)
Supplement: S1 Table — (PDF) [file ppat.1006532.s002.pdf]

S1 Table: List of genes whose expression changed at least 3 fold after infection with either S. Typhimurium or S. Typhi.

GeneID	Tm	Ty	Tm	Ty
A4GALT	1.5	2.0	3.6	1.8
ABLIM2	1.1	0.9	4.6	1.7
ABTB2	1.5	5.2	1.6	2.5
ACHE	1.4	1.7	3.8	1.3
ACSL5	0.8	1.2	1.4	3.7
ADAMTSL4	1.8	1.8	5.5	1.1
ADM	2.5	6.4	4.1	5.1
AFAP1L1	0.9	1.1	3.3	2.4
AGT	3.7	1.1	88.1	1.0
AK4	1.3	1.1	3.5	2.1
AKAP12	1.7	2.6	3.3	3.9
AKAP2	2.0	3.3	4.9	1.7
ANGPTL4	2.0	1.3	16.2	3.4
ANKRD37	1.5	3.2	3.4	3.3
APOL1	1.0	1.0	4.4	1.5
APOL2	1.4	1.2	5.4	2.8
APOL6	2.0	1.3	11.1	4.5
ARC	1.5	11.7	1.1	1.9
ARID5A	2.6	1.6	7.6	1.8
ARNTL2	1.2	1.4	3.6	2.8
ARRDC3	2.4	1.3	4.3	2.5
ATF3	1.9	4.3	2.4	1.8
ATP8B4	1.2	0.9	4.6	1.0
B3GNT3	0.8	1.0	4.1	2.7
BATF3	1.4	1.5	12.6	2.2
BCL2A1	1.0	3.6	0.8	1.6
BCL3	3.0	2.2	4.1	2.2
BCL6	1.4	1.1	3.0	0.9
BCR	1.3	1.2	3.3	1.4
BHLHE40	1.7	1.8	4.2	2.6
BIRC3	1.5	4.5	2.7	6.2
BNIP3	1.0	0.9	3.0	1.7
C10orf10	2.3	1.0	6.9	0.8
C11orf86	0.9	2.3	1.4	4.4
C11orf96	2.1	3.8	9.2	1.2
C12orf34	1.1	4.7	1.1	0.7
C1QTNF1	0.9	2.7	3.1	5.0
C1R	1.2	1.0	5.1	1.1
C20orf141	1.0	1.0	3.4	1.0

C2CD4A	1.6	1.5	10.9	0.8
C3orf52	1.2	3.1	1.8	1.8
C8orf4	3.1	2.6	2.0	3.2
CA12	1.4	1.6	4.7	2.5
CA9	1.1	0.9	8.5	3.7
CCDC68	1.3	1.3	1.9	4.3
CCDC71L	2.3	2.3	3.1	1.2
CCL2	54.9	13.1	146.5	3.0
CCL20	4.1	51.6	3.3	22.9
CCL22	1.0	3.6	1.1	3.4
CCL5	0.9	1.7	1.4	7.7
CCL7	4.5	2.6	8.8	1.0
CCRN4L	1.6	3.0	2.1	1.8
CD180	1.0	1.0	4.9	1.0
CD7	2.2	2.4	17.5	1.2
CDCP1	1.4	3.6	4.9	5.3
CEACAM1	1.0	1.0	12.3	1.5
CEBPD	6.9	5.3	7.4	1.8
CHAC1	3.1	1.6	1.8	1.0
CHST11	1.2	1.2	3.2	1.8
CHST7	1.0	3.0	1.0	1.8
CISH	3.4	1.1	5.7	1.2
CNTNAP1	0.9	1.2	3.4	2.5
CPA4	1.0	1.6	1.5	4.2
CRISPLD2	2.3	2.5	3.6	1.4
CSRNP1	3.5	3.6	8.3	2.0
CTGF	2.5	4.2	1.4	1.2
CTSB	1.0	1.1	4.2	1.0
CTSS	1.2	1.2	2.4	5.6
CXCL1	3.0	9.0	1.5	3.1
CXCL10	0.9	1.1	3.4	8.0
CXCL11	1.6	0.8	0.9	3.1
CXCL2	15.8	61.6	14.6	7.5
CXCL3	5.4	24.1	5.3	3.8
CYR61	2.0	11.6	1.1	2.3
DDX58	1.2	1.2	3.2	6.9
DKK1	1.2	3.1	1.2	3.7
DRAM1	1.3	2.1	3.5	3.5
DTX3L	1.8	1.1	3.3	2.3
DUSP1	1.9	4.4	2.4	2.2
DUSP10	0.8	3.1	0.7	1.5
DUSP5	2.9	5.1	7.5	5.5
DUSP6	1.8	2.5	4.7	3.0

EDIL3	0.8	0.9	3.4	1.5
EDN1	0.9	4.2	0.5	1.1
EDN2	0.9	5.7	0.9	0.9
EFNA1	2.3	2.8	7.4	2.3
EFNA3	1.0	1.4	6.1	2.3
EGR1	6.6	34.2	1.8	2.6
EGR3	1.2	3.2	1.0	1.1
EGR4	1.2	3.1	1.2	1.6
EID3	1.3	3.5	0.8	1.6
ELOVL2	1.3	1.2	4.1	1.9
ELOVL7	1.2	3.0	0.9	1.2
ENO2	0.8	1.3	3.6	2.3
EPAS1	1.6	1.2	7.1	2.4
EPGN	1.4	2.3	5.4	3.0
EPHA2	2.0	3.9	2.4	3.0
EREG	1.5	3.9	3.1	4.6
ERRFI1	1.6	3.0	2.7	3.1
F2RL1	5.1	1.4	7.9	1.1
F3	1.5	4.6	4.1	4.5
FAM167B	0.6	1.2	3.1	1.1
FAM212A	1.3	0.8	3.2	1.2
FAM46C	2.4	0.5	5.2	0.4
NFKBIZ	2.3	3.1	2.0	1.8
FAM70B	0.7	0.8	3.6	1.5
FAM83A	2.1	1.2	7.2	2.1
FAM83B	2.1	2.5	5.1	1.7
FBN2	1.2	1.1	3.3	1.1
FGA	11.0	1.1	247.1	1.0
FGB	5.0	1.0	231.6	1.2
FGG	24.8	0.9	670.1	1.0
FGL1	1.1	1.0	3.3	1.0
FLRT3	2.2	1.6	4.8	1.6
FOS	3.4	13.5	10.2	2.6
FOSB	1.9	18.2	1.3	1.5
FOSL1	1.8	17.0	6.4	9.3
FRMD4B	2.2	1.9	5.8	1.3
G0S2	1.2	3.8	2.8	0.9
GADD45A	1.2	3.1	0.7	1.0
GADD45B	4.5	5.5	5.3	1.4
GALNT2	1.3	1.4	3.2	1.6
GBP1	1.4	2.6	4.0	11.3
GBP3	1.4	1.8	9.0	10.0
GCNT3	3.6	1.7	4.4	1.1

GEM	2.5	9.3	2.3	1.3
GFPT2	2.0	4.2	3.4	4.3
GJA1	1.0	0.9	4.1	1.4
GLIPR1	1.9	3.4	2.8	3.9
GLRX	1.2	1.1	4.2	1.5
GPR126	1.7	1.3	9.3	2.6
GPR3	0.9	3.0	2.2	1.8
GPRC5A	1.6	1.7	4.9	3.8
GRAMD1B	2.4	2.5	4.9	2.2
GSDMC	1.2	0.9	6.5	1.1
HAPLN3	1.3	1.1	3.4	1.4
HCAR2	1.7	1.1	10.8	1.3
HCAR3	1.6	1.2	7.8	1.4
HEG1	1.6	1.0	8.1	0.9
HIF1A	1.7	1.0	3.2	1.4
HIST1H2AE	3.5	0.9	0.9	0.7
HIST1H3G	0.8	3.5	0.2	1.6
HIST1H4E	8.5	1.1	0.8	1.2
HIVEP2	1.3	1.9	3.1	1.6
HK1	1.4	1.2	4.9	2.2
HK2	2.7	1.4	8.9	2.6
HRH1	3.6	1.7	5.5	2.0
HSPA6	2.3	2.2	3.5	1.2
HSPB8	1.4	3.5	0.9	0.8
HTR3A	1.5	1.2	3.5	1.0
ICAM1	3.4	10.7	6.4	6.2
ID1	3.5	1.1	11.5	4.3
IDO1	1.1	1.0	5.5	1.1
IFI16	3.0	1.0	7.8	1.9
IFI27	2.4	0.6	0.9	3.3
IFI44	0.5	1.1	2.0	6.3
IFIH1	0.7	1.2	1.8	4.5
IFIT1	1.0	0.7	4.0	8.4
IFIT2	0.8	1.4	5.6	16.6
IFIT3	1.2	1.2	5.8	8.8
IFITM1	1.1	1.2	3.3	1.9
IGFBP1	2.0	1.5	46.8	3.5
IGFBP3	1.0	1.1	3.8	1.4
IGFL1	4.0	5.4	4.8	9.7
IKBKE	0.8	1.6	1.4	3.3
IL11	0.8	3.2	2.2	4.6
IL19	0.9	1.0	3.2	1.2
IL1A	2.1	7.4	2.6	8.6

IL1B	1.0	4.2	2.5	7.0
IL1R1	3.2	1.4	5.7	2.0
IL1RAP	1.6	1.6	4.0	2.1
IL24	2.2	1.1	71.0	3.2
IL27RA	1.7	1.8	2.1	3.2
IL2RG	1.1	1.5	2.3	4.1
IL32	1.7	5.1	5.5	15.0
IL4R	2.8	2.4	6.0	3.2
IL6	1.4	9.4	3.7	2.6
IL7R	1.7	3.0	1.9	7.2
IL8	10.5	122.3	9.7	46.6
IRAK2	1.4	3.3	2.2	3.8
IRF1	4.8	5.6	4.7	4.7
IRF9	1.5	1.2	3.6	5.1
ISG15	1.4	3.2	3.1	5.8
ISG20	2.2	2.4	7.5	2.0
ITGA2	2.5	2.4	12.7	5.2
ITGA5	1.1	2.4	3.1	2.8
ITGB3	1.0	1.0	8.6	1.0
JHDM1D	1.5	1.5	4.5	1.1
JUN	5.2	15.1	5.7	3.7
JUNB	4.7	3.9	8.7	3.3
KCTD12	2.3	1.7	3.7	1.4
KDM6B	1.2	3.2	2.1	1.5
KIR2DL5A	1.0	1.0	3.2	2.2
KLF2	0.7	3.5	2.5	1.1
KLF6	3.8	6.4	4.2	3.1
KRT16	3.4	2.2	9.1	1.8
KRT17	3.1	3.8	6.8	3.9
KSR1	1.2	1.2	3.2	1.2
LACTB	1.5	1.4	3.0	2.0
LAMP3	0.9	1.1	3.0	2.3
LBP	1.7	1.0	37.3	1.0
LIF	1.1	4.3	1.4	3.8
LINC00163	1.6	4.3	1.4	2.4
LOC100131726	3.5	1.3	3.3	2.6
LOC100288432	0.6	2.6	3.6	0.9
RBKS	2.1	1.8	3.2	1.8
LOC100499467	2.8	2.0	3.3	2.1
LOC100506540	4.0	1.3	0.8	2.1
LOC646626	1.7	3.6	1.3	2.0
LRG1	6.5	1.3	45.3	4.2
LY96	1.0	1.0	3.0	1.0

MAFB	3.2	2.3	2.2	0.8
MAFF	1.7	3.2	4.5	3.7
MAFK	1.5	2.1	3.8	2.0
MAP3K8	1.5	1.7	4.2	1.4
MDFI	1.6	1.6	3.3	2.0
METRNL	1.5	1.6	3.1	1.3
MFSD2A	1.2	3.4	1.2	1.5
MICAL2	1.7	2.0	2.2	3.2
MPZL2	1.1	1.2	3.3	1.7
MT1X	2.9	1.2	8.0	2.3
MT2A	1.3	2.1	3.5	2.9
MUC1	1.1	1.6	3.3	2.7
MUC13	2.7	2.4	10.9	2.8
NAMPT	1.7	1.1	4.1	1.3
NCOA7	3.0	1.7	5.7	1.9
NDRG1	0.8	1.1	3.3	3.2
NEDD9	1.1	3.2	1.1	2.3
NFE2	2.3	0.9	9.2	1.4
NFIL3	4.1	2.1	4.7	1.2
NFKB2	1.1	1.8	2.8	5.1
NFKBIA	2.2	5.2	1.8	2.4
NFKBID	0.9	3.6	1.4	2.5
NFKBIE	1.4	2.7	2.1	3.4
NKX2-8	1.0	3.8	1.0	1.8
NNMT	4.4	1.6	8.1	2.2
NR1D1	1.1	1.4	3.4	1.9
NR4A1	1.4	4.7	1.0	1.9
NUAK2	1.5	3.9	1.2	2.1
OASL	1.0	1.5	7.9	10.8
OBFC2A	3.5	2.3	4.7	1.6
OLR1	1.2	8.8	3.3	5.7
OSMR	2.0	1.6	6.2	2.3
PAPSS2	0.9	1.0	3.6	1.9
PARP9	1.6	0.9	3.4	1.8
PCDH1	1.7	1.3	4.1	1.4
PCDH7	2.5	3.0	4.6	2.7
PCNA-AS1	0.9	5.1	1.0	1.0
PDP1	2.4	1.5	3.5	1.6
PDZD2	1.6	1.3	5.4	2.6
PFKFB3	1.6	1.5	3.2	1.4
PFKFB4	1.1	1.4	4.0	2.1
PHLDA1	1.9	2.8	3.8	3.6
PHLDA2	1.3	2.4	2.2	3.1

PI3	1.6	2.2	1.5	3.3
PITPNC1	1.2	0.7	7.7	2.4
PLAUR	1.4	2.7	5.3	4.1
PLEKHA4	1.7	1.1	2.9	3.3
PLIN2	1.4	1.1	2.8	3.6
PLOD2	1.1	0.9	3.2	1.6
PLSCR1	1.8	1.0	5.6	1.4
PPFIA4	0.8	0.9	5.7	2.8
PPP1R15A	1.4	4.6	1.5	1.8
PPP1R3B	2.0	1.5	6.2	1.8
PRG4	4.2	1.0	12.4	0.8
PRIC285	1.2	1.4	4.4	5.1
PRSS22	4.4	2.8	2.7	0.8
PTGS2	3.9	10.5	1.4	3.4
PTPRE	2.4	1.4	4.5	1.5
PTX3	1.1	2.6	1.3	3.4
QRFP	3.1	1.6	2.6	2.0
RAB20	1.4	1.2	3.8	1.6
RAB31	1.2	1.0	3.5	1.3
RAPGEF4	1.0	0.8	5.5	0.9
RASD1	2.1	4.4	9.2	3.2
RASL11A	2.2	1.0	7.4	0.5
REEP1	0.9	1.0	3.3	1.3
RELB	1.2	2.0	4.0	6.0
RGS16	3.8	1.3	23.9	1.7
RGS2	2.9	1.3	6.2	2.5
RHEBL1	0.5	1.3	1.7	3.8
RHOB	1.6	3.1	2.3	1.3
RHOQ	1.2	1.2	4.4	1.4
RND1	1.3	0.9	17.1	2.2
RND3	2.0	3.0	1.6	1.5
RNF223	0.7	5.1	3.6	1.6
RNF24	2.0	1.4	3.8	1.2
ROM1	2.5	1.3	4.6	0.6
RPL41	5.0	0.9	4.3	1.0
RPS27	0.3	4.5	0.4	0.9
S100A3	0.8	2.9	2.1	3.3
S100P	1.3	1.3	5.3	1.4
S1PR1	3.8	1.7	7.9	1.0
SAA1	4.0	4.9	33.7	6.1
SAA2	5.7	1.7	100.3	4.1
SAMD4A	1.1	1.6	5.5	2.7
SAT1	1.7	2.4	3.4	2.5

SBNO2	1.9	1.8	4.5	1.9
SDC4	1.9	3.3	1.7	3.1
SDR16C5	1.5	1.5	10.2	4.7
SECTM1	4.2	1.5	33.8	1.4
SEMA4B	2.7	2.7	5.7	2.7
SERPINB1	1.9	1.4	9.7	1.6
SERPINB3	10.6	2.7	175.0	5.8
SERPINB4	4.5	1.5	104.0	3.0
SERPINB8	1.3	1.8	3.5	2.1
SERPINE2	1.5	2.7	2.2	3.2
SERTAD1	1.3	2.1	4.1	2.8
SH2D1B	0.9	1.4	2.9	4.1
SH3BP2	0.9	1.0	3.9	1.5
SH3TC1	2.3	2.1	3.9	1.5
SHB	1.4	3.5	3.4	2.6
SLC1A3	1.3	1.2	4.5	2.1
SLC22A4	1.2	1.0	3.1	2.0
SLC2A1	1.0	2.3	3.3	2.7
SLC2A3	1.1	1.0	8.2	1.2
SLC39A14	1.5	1.8	3.6	1.5
SLC6A15	1.0	0.8	3.2	1.2
SLCO4A1	1.0	1.5	3.7	2.5
SMOX	1.5	3.1	2.6	2.1
SNTB2	1.3	1.0	3.1	0.7
SOCS1	3.7	4.5	20.7	1.9
SOCS2	3.5	1.9	11.7	2.1
SOCS3	4.1	1.5	7.3	1.0
SOD2	2.3	4.4	8.0	6.3
SOWAHC	2.9	1.5	3.9	1.9
SPHK1	1.5	3.4	2.5	3.7
SPINK1	2.5	2.1	5.4	2.7
SPINK13	3.1	1.6	3.0	1.5
SQRDL	1.0	1.4	3.2	2.7
ST5	1.1	0.9	3.5	0.9
STAMBPL1	1.3	1.2	2.6	3.7
STAT3	1.5	1.4	3.1	1.7
STEAP4	2.2	1.3	5.6	1.2
STMN3	1.5	1.4	3.0	1.1
STOM	2.6	0.8	6.4	0.8
STX11	1.4	3.6	1.8	2.3
TGM2	1.7	1.3	8.1	3.5
THAP2	1.5	3.9	1.5	1.5
THBD	1.9	1.4	3.6	1.5

TMC5	1.4	0.7	3.1	1.1
TMEM158	1.2	3.5	1.9	2.8
TMEM173	1.1	1.1	5.1	1.4
TMEM2	2.1	1.8	6.2	1.7
TMX3	1.7	0.9	3.2	1.2
TNC	1.3	2.8	5.1	2.4
TNFAIP2	1.0	3.1	1.5	2.6
TNFAIP3	3.6	16.8	2.1	5.4
TNFAIP6	1.0	4.3	2.1	2.3
TNFRSF10D	1.8	1.5	5.1	1.9
TNFRSF12A	1.4	2.1	2.5	3.7
TNFRSF18	0.8	3.6	1.1	1.0
TNFRSF21	1.4	1.7	5.1	2.8
TNFSF15	0.8	1.0	2.0	3.1
TNIP1	0.9	2.0	1.9	3.9
TNIP2	1.3	1.6	3.7	2.1
TNS4	1.5	2.0	5.2	3.5
TRAF1	1.3	3.9	1.1	2.9
TRIB1	1.8	4.6	3.4	2.0
TSLP	1.3	3.6	0.6	1.2
TUBB3	5.8	2.2	16.8	1.6
UAP1	1.3	1.4	3.6	1.9
UBASH3B	1.5	1.5	3.6	2.3
UGCG	1.8	1.6	4.7	2.3
VLDLR	1.1	0.8	5.4	3.0
VTN	1.3	1.3	5.5	1.1
WARS	1.6	1.3	3.7	2.2
WWC1	1.5	1.5	5.8	2.3
ZC3H12A	1.3	4.0	2.8	2.1
ZFP36	6.4	9.2	20.4	2.8
ZFYVE28	1.2	0.9	5.0	1.0
ZNF503	1.3	1.3	3.4	1.1
