# Supplementary material for: Salmonella enterica serovar-specific transcriptional reprogramming of infected cells
Source: PLoS Pathog. 2017 Jul 24;13(7):e1006532. doi: 10.1371/journal.ppat.1006532 (PMC5549772; doi:10.1371/journal.ppat.1006532)
Supplement: S2 Table — (PDF) [file ppat.1006532.s003.pdf]

**S2 Table: List of strains used in this Study**

| Strain | Genotype |
| --- | --- |
| | |
| *Salmonella Typhi:* | |
| ISP2825 | wild type |
| SB2174 | *ΔinvA* |
| SB2175 | *ΔviaBC* |
| SB1843 | *ΔcdtB ΔpltAB Δsty1887 Δ1889* |
| SB2171 | *Δsty1413* |
| SB1959 | *Δsty1076* |
| SB1960 | *Δsty1360* |
| | |
| *Salmonella Typhimurium:* | |
| *SB300* | *Wild type* |
| *SB136* | *ΔinvA* |
