# Supplementary material for: Salmonella enterica serovar-specific transcriptional reprogramming of infected cells
Source: PLoS Pathog. 2017 Jul 24;13(7):e1006532. doi: 10.1371/journal.ppat.1006532 (PMC5549772; doi:10.1371/journal.ppat.1006532)
Supplement: S3 Table — (PDF) [file ppat.1006532.s004.pdf]

S3 Table: List of plasmids used in this Study

Plasmid	Description
pSB5338	$pWSK^{lacZ}$-*avrA*$_{LT2}$
pSB5345	$pWSK^{lacZ}$-*gogB*
pSB5341	$pWSK^{lacZ}$-*gtgA*
pSB4136	$pWSK^{lacZ}$-*gtgE*
pSB5343	$pWSK^{lacZ}$-*slrP*
pSB5340	$pWSK^{lacZ}$-*sopA*
pSB4830	$pWSK^{lacZ}$-*sopD2*
pSB5339	$pWSK^{lacZ}$-*sopE2*
pSB5580	$pWSK^{lacZ}$-*spvB*
pSB5581	$pWSK^{lacZ}$-*spvC*
pSB5346	$pWSK^{lacZ}$-*sseI*
pSB5347	$pWSK^{lacZ}$-*sseJ*
pSB5348	$pWSK^{lacZ}$-*sseK1*
pSB5349	$pWSK^{lacZ}$-*sseK2*
pSB5350	$pWSK^{lacZ}$-*sseK3*
pSB5344	$pWSK^{lacZ}$-*sspH1*
pSB5582	$pWSK^{lacZ}$-*steA*
pSB5342	$pWSK^{lacZ}$-*steB*
