# Supplementary material for: Salmonella enterica serovar-specific transcriptional reprogramming of infected cells
Source: PLoS Pathog. 2017 Jul 24;13(7):e1006532. doi: 10.1371/journal.ppat.1006532 (PMC5549772; doi:10.1371/journal.ppat.1006532)
Supplement: S4 Table — (PDF) [file ppat.1006532.s005.pdf]

S4 Table: qRT-PCR primers used in this study

Gene	Forward Primer	Reverse Primer
EGR1	ACCTGACCGCAGAGTCTTTTC	GCCAGTATAGGTGATGGGGG
GAPDH	CTTGAGGCTGTTGTCATACTTC	GTCCACTGGCGTCTTCAC
IL-8	TCTCAGCCCTCTTCAAAAACTTCTC	ATGACTTCCAAGCTGGCCGTGGCT
SerpinB3	CGGTCTCGTGCTATCTGGAG	ATCCGAATCCTACTACAGCGG
TTP	GACTGAGCTATGTCGGACCTT	GAGTTCCGTCTTGTATTTGGGG
